# Comparison of two dosimetric systems for quality assurance in a clinical treatment scenario for brain radiotherapy: Diode array and polymer gel dosimetry

**DOI:** 10.1002/acm2.70272

**Published:** 2025-10-10

**Authors:** Angeliki Ntouli, Georgios Kalaitzakis, John Stratakis, Kostas Perisinakis, Stefanos Kachris, Maria Tolia, Michalis Mazonakis, Thomas G. Maris

**Affiliations:** ^1^ Department of Medical Physics Medical School University of Crete Heraklion Greece; ^2^ Department of Radiation Oncology University General Hospital of Heraklion Heraklion Greece; ^3^ Department of Medical Physics University General Hospital of Heraklion Heraklion Greece

**Keywords:** brain radiotherapy, diode array, MRI, polymer gel dosimetry, Quality Assurance, SIB – VMAT, VMAT

## Abstract

**Background:**

Advanced radiotherapy techniques such as single‐isocenter volumetric modulated arc therapy (VMAT) and simultaneous integrated boost (SIB‐VMAT) require precise quality assurance (QA) due to their complexity and sensitivity to geometric and dosimetric uncertainties, especially for multi‐target configurations.

**Purpose:**

To evaluate and compare two dosimetric systems—a diode array and a polymer gel dosimeter—for quality assurance in single‐isocenter multi‐target VMAT and SIB‐VMAT plans through 3D gamma index and statistical agreement analysis.

**Methods:**

A diode array system (Delta⁴—Scandidos, Uppsala, Sweden) and a 3D printed phantom (Prime—RTsafe, Athens, Greece) embedded with in‐house polymer gel were utilized. The treatment plans for VMAT and SIB‐VMAT were created using the Monaco treatment planning system (TPS) and irradiations were performed with the Elekta Infinity linear accelerator with a 6‐MV photon beam on both Prime and Delta⁴. Analyses of the irradiated gels were performed using a 1.5T clinical MRI system. Additionally, 3D gamma indexes and Bland‐Altman analyses were conducted to evaluate the agreement between relative doses from MRI‐derived gel data and diode array's detector measurements.

**Results:**

Diode array system achieved gamma passing rates (GPRs) >99%, while polymer gel showed >95% GPR for both irradiation plans. Bland–Altman analysis indicated minimal bias (mean difference: 0.1%) and narrow limits of agreement (−1.9% to 2.2%), confirming good consistency between the two dosimetric methods for both plans delivered.

**Conclusions:**

Acceptable agreement between the two systems was observed. Both demonstrated complementary capabilities, making both essential tools for ensuring precision in advanced radiotherapy techniques. The polymer gel system offers more detailed insights compared to the diode array method, showing increased sensitivity in challenging cases that involve small targets at greater distances from the isocenter.

## 1 INTRODUCTION

Volumetric Modulated Arc Therapy (VMAT) has transformed modern radiotherapy by enabling precise dose delivery to complex target geometries while minimizing exposure to healthy tissues. This technique has proven particularly valuable for treating multiple metastases and implementing simultaneous integrated boost (SIB) strategies.^1^, ^2^ Recent studies confirm VMAT effectiveness in reducing treatment time while maintaining dosimetric precision.^3^ However, the accuracy of these sophisticated techniques relies heavily on rigorous Quality Assurance (QA) processes, which validate both relative and absolute dose calculations before clinical implementation, particularly in complex cases such as multi‐metastatic irradiation.^4^, ^5^ This is especially critical for multi‐target, single‐isocenter plans, which introduce additional dosimetric complexities due to the use of off‐axis fields and the need to accurately deliver dose to spatially separated targets from a single setup.^6^, ^7^

Various dosimetric tools have been developed to address modern radiotherapy QA requirements.^8^, ^9^, ^10^, ^11^ Ionization chambers remain the gold standard for absolute dose measurements due to their high accuracy and stability.^9^ Diode detectors offer high spatial resolution for relative dose verification in high‐gradient regions, with recent advancements in 3D diode arrays improving detection capabilities in heterogeneous geometries.^12^, ^13^, ^14^ Radiochromic films provide high‐resolution two‐dimensional (2D) dose mapping but require meticulous calibration and handling.^15^ Polymer gel dosimeters offer the unique advantage of capturing both 2D and 3D spatial dose distributions, making them superior to many conventional dosimetry systems.^15^, ^16^ Additionally, several studies confirm polymer gel dosimetry as a viable and precise QA tool for validating advanced radiotherapy techniques and how changes in chemical composition and polymerization mechanisms have influenced their dosimetric properties, accuracy, and stability for use in radiation therapy dosimetry.^10^, ^17^, ^18^, ^19^, ^20^, ^21^, ^22^, ^23^

Several studies have investigated the comparative performance of dosimetric QA approaches, highlighting their respective strengths and limitations.^24^, ^25^, ^26^ Some initial research evaluated and compared the dosimetric performance of EDR2 film and three other commercially available quality assurance devices, including IBA I'MatriXX array, PTW Seven29 array, and the Delta⁴ array, for IMRT and RapidArc verification.^27^ These studies revealed variations in error detection sensitivity and clinical feasibility, which reinforced the necessity of multi‐modal validation approaches. More recent studies have assessed the performance of diode arrays and film dosimetry in stereotactic radiotherapy (SRT), showing that high‐resolution detectors provide superior agreement in steep dose gradient regions compared to standard diode arrays, which are limited by their detector spacing and sampling resolution.^28^, ^29^, ^30^

Polymer gel dosimetry has been explored as an alternative volumetric QA tool, particularly for complex dose distributions. It has also been assessed for 4D dose verification for VMAT lung treatments, comparing its performance with ArcCHECK under motion conditions.^31^ Moreover, polymer gel dosimetry has been validated for stereotactic radiotherapy QA, comparing its performance to Delta⁴ in phantom‐based studies.^30^ A similar comparative study^32^ has examined three dosimetric systems—a 2D diode array, a radiochromic film system, and a radiosensitive polymer gel dosimeter—for intensity‐modulated arc therapy (IMAT) verification in prostate cancer treatment plans. This study demonstrates that all three methods provided acceptable verification results, each with distinct advantages. However, differences in technique, equipment, and clinical application, including abdomen plans and a 2D gamma index comparison,^32^ distinguish it from the present work. To the best of our knowledge, no research has directly compared polymer gel dosimetry with diode array systems for single‐isocenter multi‐target VMAT using realistic clinical treatment plans in cranial applications.

The aim of the study was to evaluate and compare the measurements between a 2D diode array dosimetric system (Delta⁴—Scandidos, Uppsala, Sweden) and an in‐house 3D polymer gel dosimeter within a 3D printed head phantom (Prime) in terms of 3D gamma index and statistical comparisons. The assessment was conducted using two clinical scenario treatment plans delivered through an Elekta Infinity linear accelerator (LINAC), a single‐isocenter dual partial arc VMAT and a SIB‐VMAT plan.

## 2 METHODS

### 2.1 Planning

A 3D‐printed anthropomorphic head phantom (Prime—RTsafe PC, Athens, Greece) containing an empty glass cylinder was filled with water for CT imaging. Planning images were obtained using a 256‐slice computed tomography scanner (Revolution HD, GE Healthcare, Waukesha, WI, USA) with a slice thickness of 0.625 mm. The phantom's planning CT was performed in the treatment position. Metastatic tumors were manually contoured from the CT scan using the Monaco Treatment Planning System (TPS) (Monaco 5.11, Elekta AB, Stockholm, Sweden). To simulate a range of clinical scenarios, the tumor sizes were manually adjusted, creating target volumes ranging from 1.80 to 35.01 cm^3^ at different distances from the isocenter, as shown in Table 1. Two single‐isocenter, dual partial‐arc VMAT plans using 6 MV photons were developed. The optimization criteria for these plans focused on achieving robust dose coverage to the Planning Target Volumes (PTVs) and restricting the radiation exposure of tissues excluded from the treatment volume. These plans were as follows.

**TABLE 1:** Target characteristics for both VMAT and SIB‐VMAT treatment plans.

Treatment plans	PTV	Volumes (cc)	Distance from the isocenter (cm)
VMAT	1	15.14	0.82
	2	4.12	4.17
	3	1.80	4.50
SIB‐VMAT	Tumor	35.01	0.20
	Boost	3.94	0.23

#### 2.1.1 Multiple metastases plan

Three asymmetrically distributed targets were manually delineated with specific characteristics and distances from the isocenter. Each tumor received a prescribed dose of 20 Gy delivered using a dual partial‐arc VMAT technique.

#### 2.1.2 Simultaneous integrated boost (SIB) VMAT plan

A larger tumor (35.01 cm^3^) was delineated at the cylinder's center, with a smaller 3.94 cm^3^ inner target for boost therapy. The larger tumor was prescribed a dose of 15 Gy, while the inner target received 20 Gy using a dual partial‐arc SIB‐VMAT technique.

**FIGURE 1:**
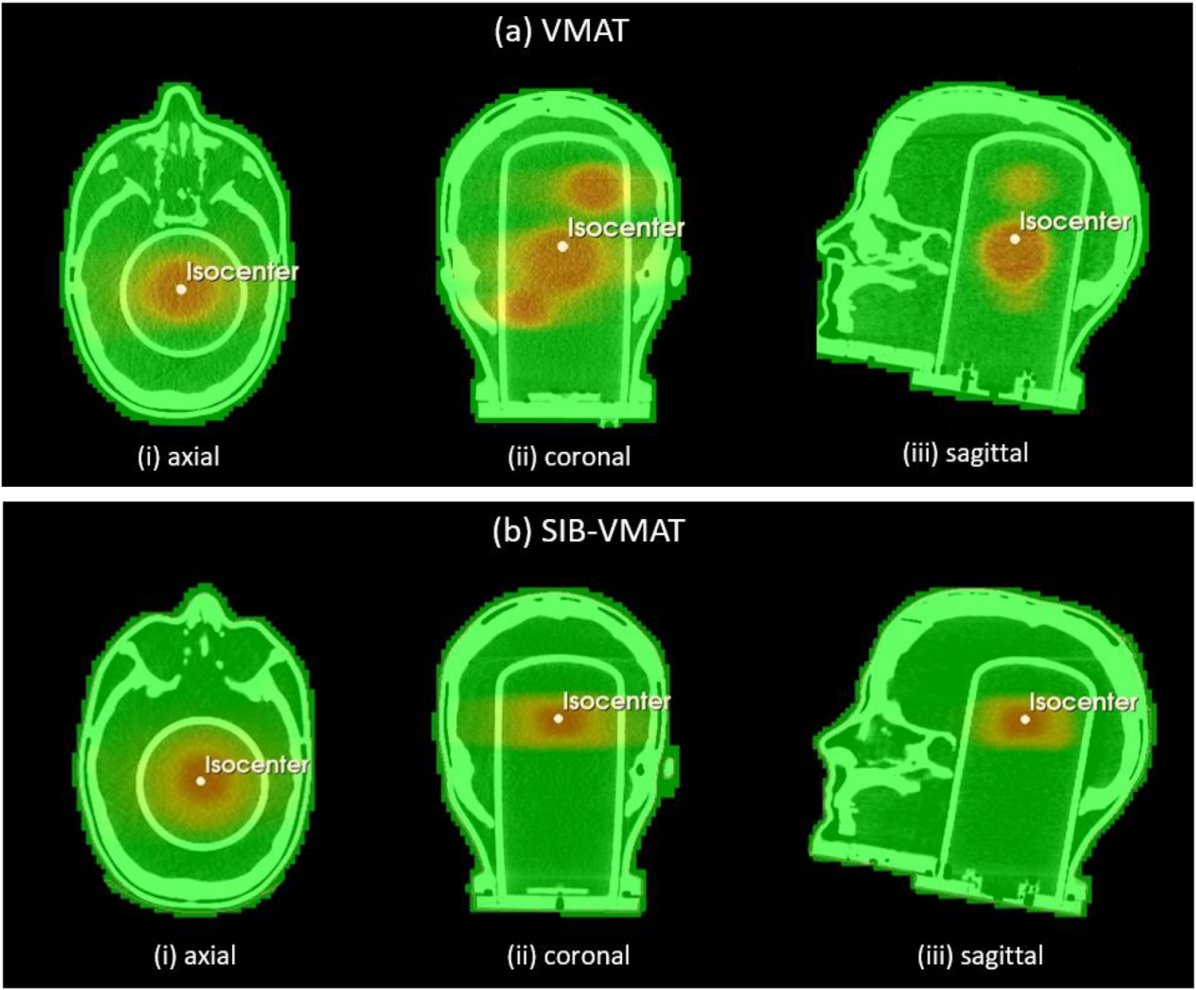
Visualization of two single‐isocenter, dual partial‐arc VMAT treatment plans overlaid on CT images of an anthropomorphic head phantom. (a) VMAT plan for multiple metastases, with three asymmetrically distributed targets positioned at varying distances from the isocenter. (b) Simultaneous Integrated Boost (SIB) VMAT plan, comprising a larger target located centrally within the phantom and a smaller, concentric inner target designated for dose escalation. For both plans, the CT images are fused with the corresponding treatment planning system (TPS) data and presented in (i) axial, (ii) coronal, and (iii) sagittal views. The isocenter is indicated in all views to illustrate its spatial relationship to the target volumes.

The prescription doses to all targets were 20 Gy in both plans. This target dose of 20 Gy ensured the reliability of dose measurements with both systems in a reasonable beam‐on time, allowing for a thorough evaluation of the dosimetric systems under clinically relevant conditions. Figure 1 illustrates the spatial distribution of the target volumes along with the corresponding isodose lines, depicting the dose distributions for each treatment plan.

### 2.2 Dosimetric systems and treatment delivery

**FIGURE 2:**
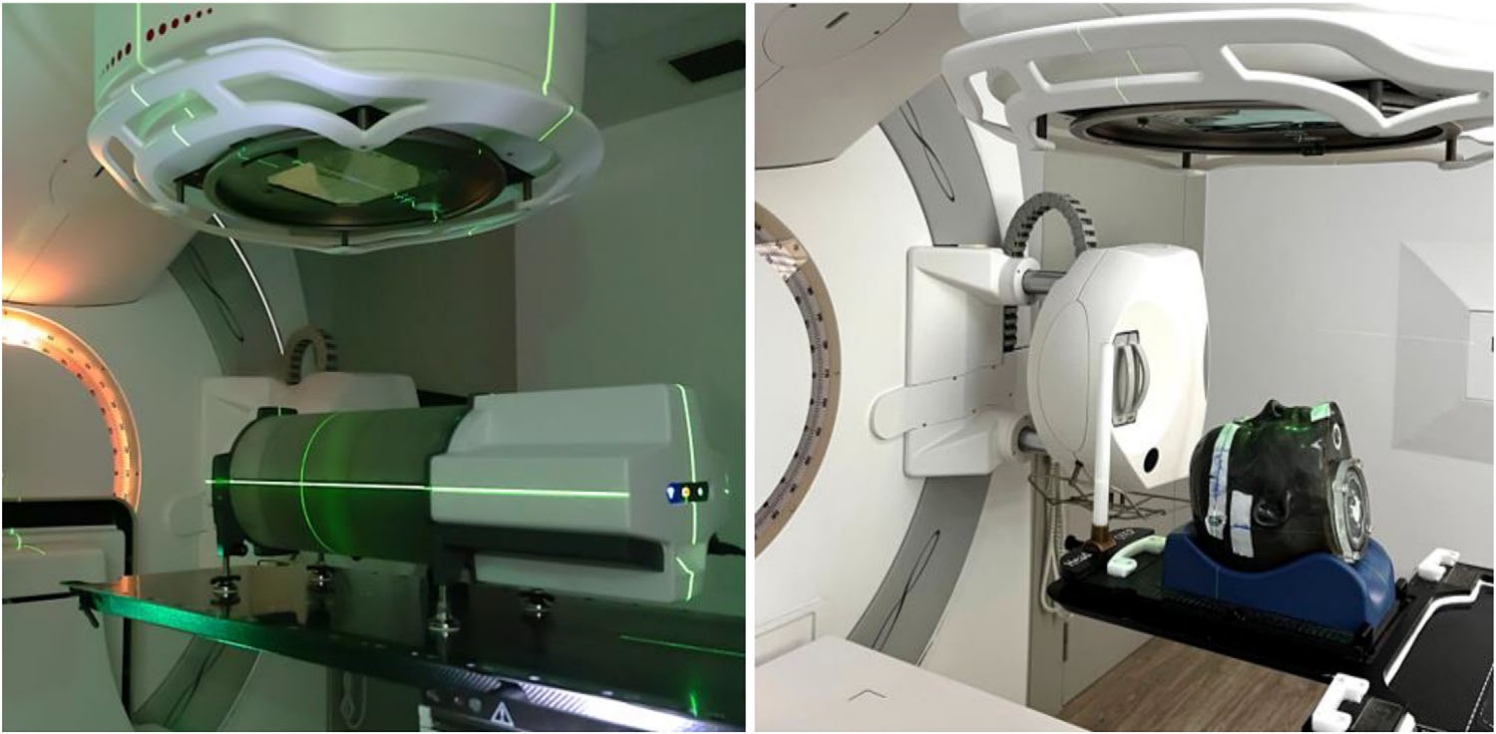
Experimental setup for dosimetric measurements using (left) the diode array system (Delta⁴) and (right) the anthropomorphic head phantom used for polymer gel dosimetry (Prime), both aligned with laser guidance on the linear accelerator for VMAT and SIB‐VMAT plan verification.

The Delta⁴ phantom (ScandiDos, Uppsala, Sweden) is a diode array‐based system designed for patient‐specific QA in VMAT and IMRT. It consists of two orthogonal planes of diode detectors arranged in a cylindrical acrylic housing, with diodes spaced at 5 mm in the central region and 10 mm in the outer regions, allowing for dose measurements in both axial and sagittal orientations. Prior to measurements, the Delta⁴ phantom was calibrated and aligned to the beam isocenter using laser guidance, following the manufacturer's guidelines. The VMAT treatment plans were first delivered to the Delta⁴ phantom for dose verification and subsequently delivered to the Prime phantom for further evaluation, as seen in Figure 2.

Prime (RTsafe, Athens, Greece), is a 3D printed head phantom based on the CT images of a real patient. For the needs of this study, it was equipped with two empty glass cylinders, one for each treatment plan. Because of its proven stability and linear dosage response, in‐house prepared normoxic N‐vinylpyrrolidone polymer gel (VIPET) dosimeters were made in accordance with an established protocol.^33^, ^34^, ^35^ The VIPET polymer gel dosimeters were manufactured with a composition including 6% w/w monomer of N‐vinylpyrrolidone (VIPE), 4% w/w cross‐linker N,N0‐methylenebisacrylamide (bis), 5% w/w gelatin, 7 mM tetrakis (hydroxymethyl) phosphonium chloride (THPC), and 85% w/w double‐distilled deionized water. Preparation was conducted under normoxic conditions to prevent oxygen‐related polymerization inhibition. After preparation, the gel was poured into the phantom's cylinders and stored overnight at ∼6°C in a dark room to solidify. Irradiation was performed 24 h post‐preparation, with the phantom acclimatized to room temperature 4 h prior to irradiation.^36^ Set up and positioning of the phantom (Prime) on the treatment couch was initially performed using its crosshairs for alignment (Figure 2). A CBCT scan was then acquired using the XVI system embedded in the LINAC and registered to a reference CT scan to refine its position and ensure accurate alignment with the treatment isocenter.

Both treatment plans were delivered using dual partial‐arc VMAT with 6 MV photons on an Infinity linear accelerator (Elekta AB, Stockholm, Sweden), with a collimator angle of 0 degrees. The gantry rotated from 240° to 120°, with one arc moving clockwise and the other counter clockwise. Each plan was delivered in a single irradiation session, ensuring consistent exposure conditions for both dosimetric systems.

### 2.3 MR image acquisition

The Prime Phantom, containing the irradiated gel‐filled cylinders, underwent MRI scans for dose readout 24 h after irradiation. All MRI measurements were conducted on a 1.5T whole‐body clinical MRI system (MAGNETOM Vision/Sonata Siemens Healthcare, Erlangen, Germany) with a high‐performance gradient system similar to that of a 3T scanner (maximum gradient strength: 40 mT/m, gradient pulse rise time: 200 µs, gradient slew rate: 200 mT/m/ms). Signal detection was accomplished with a standard two‐channel array head coil. Measurements of the Prime Phantom were made in an ambient temperature of 20°C, 24 h after irradiation.

A 2D, multi‐slice, multi‐echo, Half Fourier Single Shot Turbo Spin Echo (HASTE) PD to heavily T2‐weighted sequence was implemented with four Echo Times (TE = 36, 436, 835, 1230 ms) and TR = 2000 ms. The number of averages was set to 20 to increase signal‐to‐noise ratio, while the bandwidth was set to 780 Hz/pixel to minimize MR‐related geometric distortion.^37^ Measurements were conducted by using the following parameters: Field of View (FOV) = 175 × 350 on the transversal plane, matrix size = 256 × 128, number of slices = 77, slice thickness = 2 mm, resulting in a voxel size of 1.4 × 1.4 × 2 mm^3^, Flip Angle = 180°. The total scan time was 27 min.

### 2.4 Data analysis

For the generation of T_2_ parametric maps, a weighted linear regression analysis was applied.^37^, ^38^ Quantitative T_2_ parametric maps were generated using TESLA QMRI Utilities‐X, an in‐house software tool (designed by two of the authors T.G.M., G.K.), and integrated into the local PACS system (EVORAD) platform. T_2_ parametric maps were fused with the CT dataset using the Monaco TPS. Registration was assessed based on anatomical bone structure alignment. Following that, the R_2_ transverse magnetization relaxation rates, expressed as R_2_ = 1/T_2_, were calculated by taking the reciprocal of the T_2_ parametric maps, leveraging the linear relationship between R_2_ values and the administered dose.^39^ Using in‐house Matlab (The MathWorks, Inc., Natick, MA) routines, a 3D relative gel dose map was generated by converting R_2_ relaxation rates to dose values. The linear relationship between R_2_ relaxation rates and dose was established through a two‐point calibration method. This involved irradiating reference gels with known, uniform doses of 0 Gy (representing unirradiated gel) and 20 Gy (representing the maximum prescribed dose in our plans).^40^ The corresponding R_2_ relaxation rates of these reference gels were then measured using the MRI system. These two reference points allowed for the derivation of a linear calibration curve (R_2_ vs. dose), which was subsequently applied to convert the R_2_ maps of the experimental gels into relative dose maps. This two‐point approach is a well‐established method for relative dose comparisons in polymer gel dosimetry.^41^ Structure set data and RTDose information were transferred from the TPS to evaluate the spatial precision of radiation delivery.

The relative dose for the diode array system (Delta⁴) was obtained by normalizing the measured dose values to the maximum delivered dose, which corresponded to the prescription dose of 20 Gy, as the plans were optimized to achieve a homogeneous dose distribution within the target volume. For the polymer gel dosimeter, the R_2_ values were converted to relative dose values by normalizing to the average delivered dose. This corresponded to the prescription dose of 20 Gy, following a linear calibration derived from the two reference points at 0 and 20 Gy, respectively. Dose data from the diode array system (Delta⁴) were selected based on coordinates in the patient reference system to ensure coverage of regions of dosimetric interest as defined by the TPS. To facilitate a direct point‐by‐point comparison, data points were extracted from the MRI‐derived gel dose maps that precisely matched the physical detector positions of the Delta⁴ within the target volumes. This selection criterion was necessary to ensure a scientifically sound comparison of dose measurements at the exact same spatial locations for both systems. It is also important to note that no outliers were discounted in this analysis; all valid matched data points were included.

Following that, a 3D gamma index analysis was conducted to quantitatively assess the dosimetric agreement and spatial correlation between the measured and planned dose distributions. Phantom positioning for both Delta⁴ and Prime was performed independently using established clinical protocols, including laser guidance and CBCT (for Prime phantom), to ensure accurate alignment with the treatment isocenter prior to dose acquisition and gamma analysis. The calculation was performed based on established methodologies and gamma evaluation criteria were set to 3%/2 mm and 5%/2 mm, using in‐house Matlab routines, and the pass rate was evaluated within clinically relevant dose regions.^5^, ^42^ This procedure was repeated for both irradiation plans, ensuring precise spatial correlation and enabling a robust evaluation of dosimetric agreement across critical dosimetric regions.

### 2.5 Statistical comparison

Bland‐Altman analysis and a paired *t*‐test were conducted to assess agreement between diode array (Delta⁴) dose measurements and polymer gel's (Prime) relative doses for both treatment plans, using MedCalc statistical software, version 20.0 (MedCalc Software, Ostend, Belgium). Among the available methods for assessing agreement between two measurement techniques, Bland–Altman analysis was selected due to its focus on evaluating the consistency of differences between relative measurements rather than their correlation. Given that polymer gel dosimetry and diode array are inherently different in measurement principles, this approach was deemed more appropriate. The average difference (mean bias) between the two methods was calculated as the mean of the differences between paired measurements, while the limits of agreement (LoA) were determined as the mean difference ± 1.96 times the standard deviation of these differences, indicating the range within which 95% of the differences are expected to fall. However, it is important to note that the Delta⁴ detector array provided limited coverage of the full anatomical structures involved in the treatment plans. This constraint particularly affected the evaluation of dose distribution, as the predefined detector positions did not encompass all regions of high‐dose gradients or peripheral targets. The dose data points that were finally compared correspond to PTV2 for the VMAT plan, while for SIB‐VMAT plan dose data points represent spatial positions matching the detector array configuration within the Boost planning target volume (Table 1).

## 3 RESULTS

### 3.1 Diode array system (Delta⁴)

Gamma analysis results for different criteria using Delta⁴ phantom showed high passing rates. The gamma analysis was performed to assess the agreement between the measured dose distribution (from the Delta⁴) and the TPS calculated dose distribution, which served as the reference. For VMAT plan, using 3%/2 mm and 5%/2 mm criteria, passing rates revealed 99.5% and 100%, respectively. In SIB‐VMAT plan, both passing rate criteria showed passing rates of 100%.

### 3.2 Polymer gel (Prime phantom)

3D gamma calculations were performed to assess volumetric agreement between planned and delivered doses (Table 2). Gamma passing rates (GPRs) were calculated using dose difference/distance‐to‐agreement (DD/DTA) criteria of 3%/2 mm and 5%/2 mm to assess the dosimetric agreement between the planned and delivered dose distributions. Calculations used the same passing criteria as in Delta⁴, with a 1% low‐dose cut‐off threshold. For the multiple metastases VMAT plan, GPRs using the 3%/2 mm criterion ranged from 95.45% for the superior target (PTV 2) to 97.32% for the inferior target (PTV 3). With the 5%/2 mm criterion, GPRs improved to 97.60%–99.73%. For the SIB‐VMAT plan, the larger target (Tumor) achieved 98.30% and 99.41% passing rates with 3%/2 mm and 5%/2 mm criteria, respectively, while the smaller boost volume (boost) showed perfect agreement with 100% passing rates for both criteria.

**TABLE 2:** Gamma index passing rate results for the multiple metastases VMAT plan and the simultaneous integrated boost VMAT (SIB‐VMAT), comparing the polymer gel dosimeter (PTV‐specific rates) and the Delta⁴ diode array (global rates). The pass criteria are presented as percentage dose difference (%DD) and distance‐to‐agreement (DTA in mm).

Dosimetric system	Plan	Evaluation region	Pass criteria	Gamma passing rate (GPR)
Polymer gel	VMAT	1	3%/2 mm	96.72
		2	3%/2 mm	95.45
		3	3%/2 mm	97.32
		1	5%/2 mm	97.60
		2	5%/2 mm	98.39
		3	5%/2 mm	99.73
	SIB‐VMAT	Tumor	3%/2 mm	98.30
		boost	3%/2 mm	100
		Tumor	5%/2 mm	99.41
		boost	5%/2 mm	100
Delta^4^	VMAT	Global	3%/2 mm	99.50
		Global	5%/2 mm	100
	SIB‐VMAT	Global	5%/2 mm	100
		Global	3%/2 mm	100

### 3.3 Statistical analysis

**FIGURE 3:**
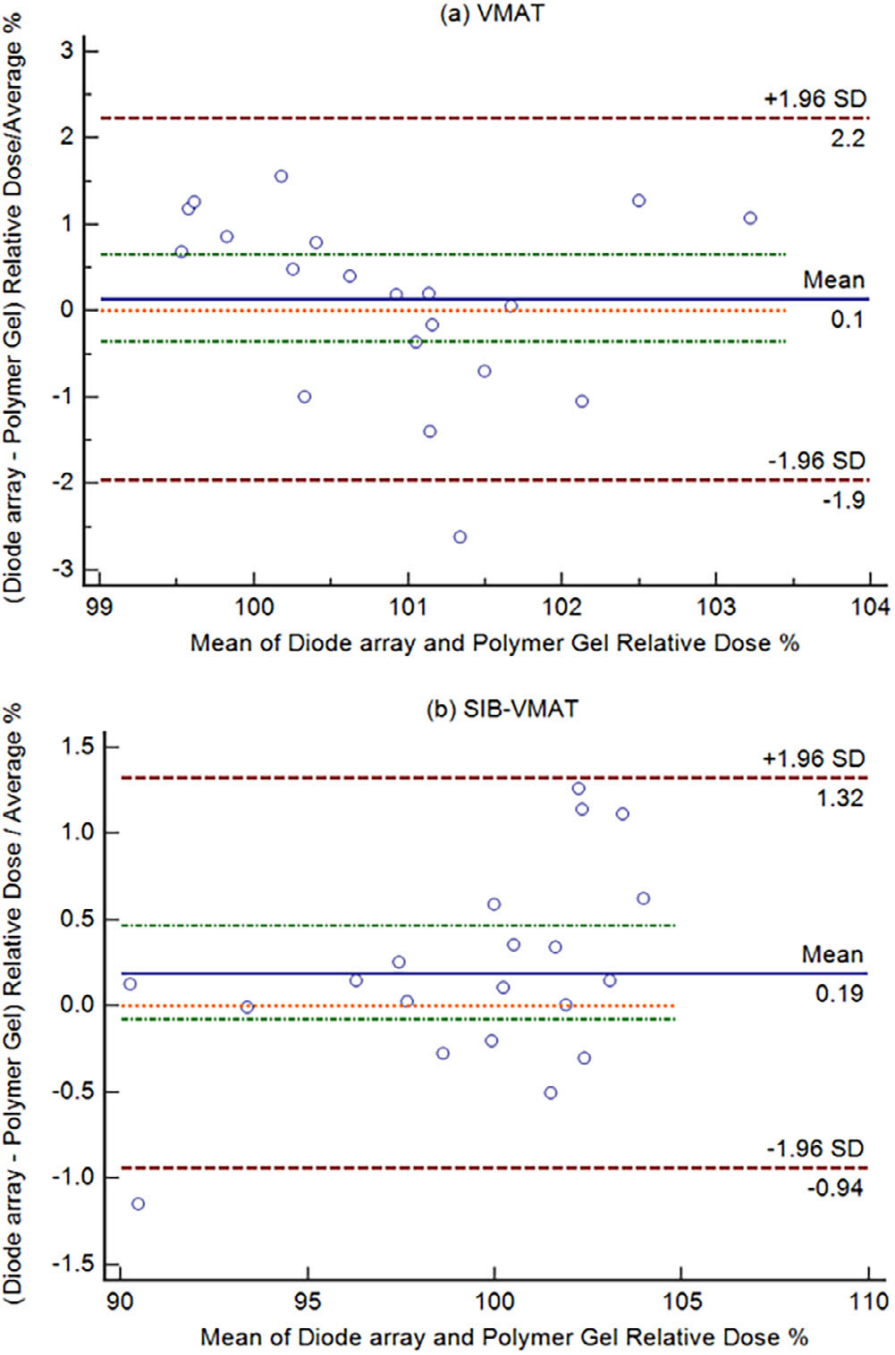
Bland‐Altman plots comparing diode array (Delta⁴) dose measurements and polymer gel's (Prime) relative doses for (a) multiple‐target VMAT plan and (b) SIB‐VMAT plan. Each point represents the difference between the two systems plotted against their average dose. The green horizontal line indicates the mean difference (bias) between the two dosimetric systems, while the yellow horizontal lines represent the 95% Limits of Agreement (mean difference ± 1.96 standard deviation of the differences), defining the range within which 95% of the differences between the two methods are expected to lie.

Paired *t*‐test results revealed no statistically significant difference between the mean measurements of the two systems (*p* > 0.05). The relative dose measurements for both treatment plans, VMAT and SIB‐VMAT, for each dosimetric system (Delta⁴ and Prime) are presented in Table 3. Bland‐Altman analysis evaluated agreement between diode array (Delta⁴) dose measurements and polymer gel (Prime) relative doses for both treatment plans. For the multiple‐target VMAT plan (Figure 3a), analysis showed minimal bias (+0.1%) with limits of agreement ranging from −1.9% to +2.2%. For the SIB‐VMAT plan (Figure 3b), analysis revealed a slightly higher bias (+0.19%) and narrower limits of agreement (−0.94% to +1.32%).

**TABLE 3:** Diode array system (Delta⁴) and Polymer Gel (Prime) relative dose measurements for VMAT and SIB‐VMAT plans. Measurements represent normalized dose values expressed as percentages of the prescription dose (20 Gy). VMAT data points correspond to PTV2 target volume locations, while SIB‐VMAT data points represent spatial positions matching the detector array configuration within the Boost planning target volume. Prime dose values were derived from R_2_ measurements using the linear calibration model described in the text.

Data point	VMAT		SIB‐VMAT	
	Delta⁴ (%)	Prime (%)	Delta⁴ (%)	Prime (%)
1	100.16	98.98	100.28	99.68
2	101.60	102.66	102.22	102.53
3	100.25	99.39	101.77	101.42
4	100.81	100.00	100.68	100.32
5	99.83	100.82	102.87	101.58
6	100.86	101.23	104.00	102.84
7	100.24	98.98	103.16	103.00
8	100.01	102.66	101.23	101.74
9	100.82	100.41	101.91	101.90
10	100.96	99.39	104.28	103.63
11	101.23	101.02	102.91	101.74
12	101.07	101.23	100.27	100.16
13	101.70	101.64	99.80	100.00
14	101.14	101.84	98.47	98.74
15	101.02	100.82	97.57	97.31
16	103.15	101.84	96.36	96.21
17	100.49	100.00	97.66	97.63
18	100.44	101.84	93.36	93.36
19	99.87	99.18	89.96	91.00
20	103.78	102.66	90.32	90.21
SD	0.98	1.21	4.09	3.80
Mean	100.97	100.83	99.45	99.25

## 4 DISCUSSION

Our results show that, minimal differences exist between the two dosimetric methods, with both demonstrating strong agreement with TPS calculations. Diode array dosimetry is a well‐established method for 2D dose verification in VMAT techniques, whereas, polymer gel dosimetry offers 3D dose mapping and high spatial resolution.^43^ However, it depends on MRI scanning and is influenced by factors such as dose rate.^44^

Both dosimetric systems in our study demonstrate strong agreement with TPS calculations, with GPRs exceeding 95% (Table 2), under 3%/2 mm and 5%/2 mm criteria for both treatment plans. A paired *t*‐test revealed no statistically significant difference between the mean measurements of the two systems (*p* > 0.05), consistent with the findings of our Bland–Altman analysis, which more directly assessed agreement and clinical interchangeability. Furthermore, the minimal bias (+0.1%) and narrow Bland‐Altman limits of agreement (−1.9% to +2.2%) confirm the strong consistency between the two methods (Figure 3). Notably, only one data point fell outside these limits, reinforcing the overall strong agreement between the two dosimetric systems. The improved agreement in SIB‐VMAT plan (Figure 3b), suggests greater dosimetric consistency in localized high‐dose regions for both dosimetric methods. Importantly, neither method exhibited systematic overestimation or underestimation of dose values in either plan, ensuring the precision required for QA radiotherapy. It is worth noting that the 3D gamma index values obtained with the gel dosimeter were slightly lower. This may be attributed to the gel's full 3D measurement capability, which can potentially reveal more discrepancies across the volume compared to diode dosimetry.

Several studies have investigated the performance of diode array and polymer gel dosimeters in radiotherapy QA. Chandraraj et al.^45^ evaluated multiple 2D dosimetric systems, including Delta⁴, for RapidArc and IMRT QA, and concluded that a combination of all could be useful. Similarly, our study suggests that both dosimetric systems, Delta⁴ and polymer gel, can complement each other and be effectively used for VMAT verification. Moreover, several commercial QA devices for stereotactic radiotherapy have been compared, highlighting the limitations of diode arrays in high‐gradient dose regions.^30^ Our findings align with their observations, as diode array system's (Delta⁴) fixed detector spacing may not fully capture dose variations, particularly in steep gradient areas. This is an issue that polymer gel dosimetry resolves through 3D dose mapping.^46^, ^47^

While the Delta⁴ system provides discrete point measurements at fixed detector locations (e.g., a total of 20 data points were used for PTV2 and Boost PTV), the polymer gel inherently captures the dose distribution throughout the entire volume of interest, thereby providing a significantly larger number of data points across all PTVs and surrounding regions (Table 3). This volumetric capability is indeed a significant advantage, particularly for small or irregularly shaped targets, as it offers a more comprehensive representation of the delivered dose. This aspect highlights the complementary nature of the two dosimetric systems, where the continuous 3D data from the polymer gel can reveal discrepancies or provide insights into dose complexities that might be missed by the discrete sampling of the diode array, especially in highly modulated or multi‐target plans.^48^, ^49^

Additionally, prior studies that compare polymer gel dosimetry with 2D diode arrays were conducted in controlled, pilot‐scale settings rather than using clinical treatment plans.^31^, ^32^ Our study extends this research by comparing the two dosimetric systems using actual clinical treatment plans, allowing for a direct comparison across different target volumes in both VMAT and SIB‐VMAT techniques. This approach enabled a thorough evaluation of their performance under realistic clinical conditions, providing deeper insights into their practical applicability for treatment verification. A similar comparative study using three dosimetric systems (MapCheck 2D diode array, BANG3 polymer gel dosimeter and Gafchromic EBT2 radiochromic film) for IMAT verification in prostate cancer treatment plans, concluded that all three systems provided acceptable verification results, each with its unique advantages.^15^ Our study extends this comparison to a clinical VMAT and SIB‐VMAT treatment plan for brain metastases. Focusing on a statistical comparison between diode array and polymer gel dosimetry within the target volume, our results demonstrated good agreement between the two methods.

Furthermore, unlike previous studies that faced challenges due to phantom size and the non‐uniform properties of the gel, led to larger dose discrepancies, our study overcame these issues by using an anthropomorphic phantom (Prime). Treatment plans were designed based on CT images of this phantom to closely replicate real clinical scenarios. This approach provided realistic anatomical representation, enabling for more accurate dose evaluation and minimizing potential distortions in dose response. With the size and geometry of a real patient, PTV localization within the polymer gel area was feasible for the treatment plan, ensuring accurate dose measurement. The use of non‐generic phantom size, also allowed for precise target delineation in the areas of interest. This allowed us to avoid distortions in dose response caused by oxygen effects and spatial limitations that can arise when PTV locations are not aligned with the phantom's shape and size. The potential for oxygen contamination during gel preparation and filling is a crucial concern in gel dosimetry.^18^, ^44^ To mitigate this risk, several measures were implemented, such as using a glass phantom insert to minimize diffusion, and strictly controlling the time interval between gel fabrication and irradiation within a 24‐h interval. All target structures were positioned centrally, well away from potential oxygen diffusion pathways, following the manufacturer's recommendations. As no visible artifacts were observed during the MRI scanning and analysis, the oxygenation effect was considered negligible for this study. Overall, this approach suggests that when gel dosimetry is used with properly designed anthropomorphic phantoms, it can enhance dose verification, particularly in complex scenarios with small targets at greater distances from the isocenter. The findings of this study, further reinforce the complementary role of polymer gel dosimetry alongside diode array dosimetry in clinical applications, particularly in verifying complex dose distributions in VMAT and SIB‐VMAT techniques, where steep gradients and multiple targets located far from the isocenter present significant dosimetric challenges.

While diode arrays remain a practical and efficient tool for routine QA, their limited spatial resolution underscores the need for volumetric verification in such cases. This efficiency of diode arrays stems from their near real‐time measurement capability. In contrast, polymer gel dosimetry, despite offering comprehensive 3D dose information, involves additional steps such as MRI scanning and post‐processing of the MRI data, introducing a longer turnaround time. For instance, polymerization and temperature equilibration typically necessitate 24 h post‐preparation before irradiation, and MRI scans for dose readout are conducted 24 h after irradiation, meaning only one irradiation per gel phantom can usually be completed per 24‐h period.^34^, ^35^ Therefore, while invaluable for complex cases with small targets or steep dose gradients, the current processing workflow of polymer gel dosimetry makes it less feasible for routine, patient‐specific QA for every patient, rendering it more suited for commissioning or specific research applications where detailed 3D dose verification is critical. Future work related to this study could involve a comprehensive comparison, incorporating diode arrays, film dosimetry and polymer gel in a realistic cranial treatment plan, also considering different error scenarios.

While this study demonstrated strong consistency between the two dosimetric systems, several limitations should be considered. The comparison was made on multiple data points, however, they were limited to selected ROIs and did not comprehensively cover the entire 3D dose distribution. A more extensive comparison, including additional PTVs or organs at risk (OARs), could improve the robustness of the findings. Furthermore, the study was conducted using a single planning system and linac model, which may limit how broadly the results apply across different equipment or planning configurations.^16^, ^43^

Despite these limitations, our work highlights a critical synergy between the two dosimetric systems, underscoring the importance of selecting QA methods based on both their practical efficiency and their dosimetric capabilities. The ability of the polymer gel to provide a comprehensive, high‐resolution 3D dose map makes it a powerful benchmarking tool for validating advanced radiotherapy techniques in complex scenarios. In contrast, the diode array serves as an efficient, near‐real‐time tool for routine QA. This work suggests that a multi‐modality approach, where gel dosimetry is used to commission and periodically benchmark the performance of the diode array in challenging cases, could lead to a more robust and comprehensive QA program. Future research could expand this work by including additional irradiation plans with varying target complexities, as well as comparisons with film dosimetry and diode array systems across a range of clinical scenarios.

## 5 CONCLUSIONS

This study demonstrates a strong agreement between a 2D array dosimeter (Delta⁴) and polymer gel dosimetric system (Prime), in realistic clinical treatment plans using single‐isocenter dual partial arc VMAT and SIB‐VMAT plans. Both methods achieved clinically acceptable GPRs, exceeding 95% in all cases, with polymer gel dosimetry providing the additional benefit of separate 3D gamma index measurements for each target. These findings suggest that integrating both dosimetric methods provides a more comprehensive and robust verification strategy for single isocenter VMAT QA in clinical practice.

## AUTHOR CONTRIBUTIONS

All authors listed in this manuscript fulfil the JACMP recommendations for authorship. *Study concepts and design*: Thomas G. Maris, Angeliki Ntouli, Michalis Mazonakis, and Georgios Kalaitzakis. *Guarantor of integrity of the study*: Thomas G. Maris and Michalis Mazonakis. *Literature research*: Angeliki Ntouli and Georgios Kalaitzakis. *Phantom study*: Angeliki Ntouli, Georgios Kalaitzakis, and Thomas G. Maris. *Clinical studies*: Michalis Mazonakis, Stefanos Kachris, John Stratakis, Μaria Tolia, and Kostas Perisinakis. *Experimental studies/data analysis*: Angeliki Ntouli and Georgios Kalaitzakis. *Statistical analysis*: Angeliki Ntouli. *Primary write up of the manuscript*: Angeliki Ntouli. *Reviewing and editing the manuscript*: All authors.

## CONFLICT OF INTEREST STATEMENT

The authors declare no conflicts of interest.

## Data Availability

The data that support the findings of this study are available from the corresponding author upon reasonable request.
